# Supplementary material for: Diet and nutrition status of adult multidrug-resistant tuberculosis cases, household controls, and community controls in Mumbai, India
Source: PLOS Glob Public Health. 2026 Jan 13;6(1):e0005778. doi: 10.1371/journal.pgph.0005778 (PMC12798980; doi:10.1371/journal.pgph.0005778)
Supplement: S1 Table — (DOCX) [file pgph.0005778.s001.docx]

**S1 Table.** Individual and household food consumption-related habits by population subgroup.

| **Question** | **Cases, N=90** | **HH controls, N=180** | **Community controls, N=82** | **Male cases, N=42** | **Female cases, N=48** | **Male HH controls, N=84** | **Female HH controls, N=96** | **Male community controls, N=37** | **Female community controls, N=45** |
| --- | --- | --- | --- | --- | --- | --- | --- | --- | --- |
| *Household consumption of fats and oils (liters per capita per month), mean (SD)* | | | | | | | | | |
| Sunflower/safflower oil | 1.3 (0.5) | 1.2 (0.5) | 1.4 (0.9) | 1.3 (0.6) | 1.4 (0.4) | 1.2 (0.5) | 1.2 (0.5) | 1.6 (1.2) | 1.2 (0.3) |
| Groundnut oil | 1.1 (0.3) | 1.2 (0.4) | 1.0 (0.4) | 1.1 (0.4) | 1.0 (0.3) | 1.2 (0.4) | 1.2 (0.5) | 1.1 (0.5) | 1.0 (0.2) |
| Coconut oil | 0.0 (0.0) | 0.0 (0.0) | 0.0 (0.0) | 0.0 (0.0) | 0.0 (0.0) | 0.0 (0.0) | 0.0 (0.0) | 0.0 (0.0) | 0.0 (0.0) |
| Palm oil | 1.1 (0.5) | 1.0 (0.4) | 1.1 (0.6) | 1.2 (0.6) | 0.9 (0.4) | 1.0 (0.3) | 1.0 (0.5) | 1.0 (0.3) | 1.2 (0.7) |
| Mustard oil | 0.8 (0.4) | 0.9 (0.6) | 1.0 (0.5) | 0.0 (0.0) | 0.8 (0.4) | 0.8 (0.7) | 0.9 (0.4) | 1.1 (0.5) | 1.0 (0.5) |
| Dalda/vanaspathi | 0.1 (0.1) | 0.1 (0.1) | 0.1 (0.1) | 0.1 (0.1) | 0.1 (0.0) | 0.1 (0.1) | 0.1 (0.1) | 0.1 (0.1) | 0.1 (0.0) |
| Olive Oil | 0.0 (0.0) | 0.0 (0.0) | 1.3 (0.0) | 0.0 (0.0) | 0.0 (0.0) | 0.0 (0.0) | 0.0 (0.0) | 0.0 (0.0) | 1.3 (0.0) |
| Corn Oil | 0.0 (0.0) | 0.0 (0.0) | 0.0 (0.0) | 0.0 (0.0) | 0.0 (0.0) | 0.0 (0.0) | 0.0 (0.0) | 0.0 (0.0) | 0.0 (0.0) |
| Rice Bran Oil | 0.0 (0.0) | 0.0 (0.0) | 0.7 (0.5) | 0.0 (0.0) | 0.0 (0.0) | 0.0 (0.0) | 0.0 (0.0) | 0.7 (0.5) | 0.0 (0.0) |
| Soya bean Oil | 0.0 (0.0) | 1.5 (0.0) | 1.1 (0.9) | 0.0 (0.0) | 0.0 (0.0) | 1.5 (0.0) | 0.0 (0.0) | 0.0 (0.0) | 1.1 (0.9) |
| Butter | 0.1 (0.0)^†^ | 0.1 (0.0)^¥^ | 0.1 (0.1)^†¥^ | 0.1 (0.0) | 0.0 (0.0) | 0.1 (0.0) | 0.0 (0.0) | 0.3 (0.0) | 0.1 (0.1) |
| Ghee | 0.1 (0.1) | 0.1 (0.1) | 0.1 (0.1) | 0.1 (0.1) | 0.1 (0.1) | 0.1 (0.1) | 0.1 (0.1) | 0.2 (0.2) | 0.1 (0.1) |
| Do you routinely remove fat/skin from meat before cooking, n (%) | 68 (75.6) | 141 (78.3) | 59 (72.0) | 31 (73.8) | 37 (77.1) | 62 (73.8) | 79 (82.3) | 24 (64.9) | 35 (77.8) |
| How many coconuts do you use for cooking in a month, mean (SD) | 3.2 (4.6) | 2.8 (3.8) | 3.8 (4.4) | 3.3 (3.8) | 3.1 (5.2) | 2.6 (4.3) | 3.0 (3.4) | 4.7 (5.9) | 3.2 (2.5) |
| How many grams of coconut powder do you use for cooking in a month, mean (SD) | 1.2 (10.5) | 2.5 (21.3) | 1.3 (11.0) | 2.4 (15.4) | 0.1 (0.7) | 0.0 (0.1) | 4.7 (29.1) | 2.8 (16.4) | 0.1 (0.5) |
| *Thickening agents added to curries or vegetables at least twice per week, n (%) (multiple responses allowed)* | | | | | | | | | |
| Coconut | 58 (64.4) | 103 (57.2) | 57 (69.5) | 25 (59.5) | 33 (68.8) | 45 (53.6) | 58 (60.4) | 22 (59.5) | 35 (77.8) |
| Groundnuts | 33 (36.7) | 64 (35.6)^¥^ | 41 (50.0)^¥^ | 16 (38.1) | 17 (35.4) | 32 (38.1) | 32 (33.3) | 16 (43.2) | 25 (55.6) |
| Roasted Bengal gram | 6 (6.7) | 14 (7.8)^¥^ | 14 (17.1)^¥^ | 4 (9.5) | 2 (4.2) | 5 (6.0) | 9 (9.4) | 7 (18.9) | 7 (15.6) |
| None | 28 (31.1) | 66 (36.7)^¥^ | 18 (22.0)^¥^ | 15 (35.7) | 13 (27.1) | 32 (38.1) | 34 (35.4) | 9 (24.3) | 9 (20.0) |
| *What type of milk do you regularly consume, n (%)* | | | | | | | | | |
| Whole milk | 8 (8.9) | 19 (10.6) | 10 (12.2) | 4 (9.5) | 4 (8.3) | 8 (9.5) | 11 (11.5) | 2 (5.4) | 8 (17.8) |
| Skimmed milk | 0 (0.0) | 3 (1.7) | 2 (2.4) | 0 (0.0) | 0 (0.0) | 2 (2.4) | 1 (1.0) | 1 (2.7) | 1 (2.2) |
| Toned milk | 83 (92.2) | 157 (87.2) | 69 (84.1) | 38 (90.5) | 45 (93.8) | 73 (86.9) | 84 (87.5) | 33 (89.2) | 36 (80.0) |
| Whole milk powder | 0 (0.0) | 0 (0.0) | 0 (0.0) | 0 (0.0) | 0 (0.0) | 0 (0.0) | 0 (0.0) | 0 (0.0) | 0 (0.0) |
| Skimmed milk power | 0 (0.0) | 1 (0.6) | 0 (0.0) | 0 (0.0) | 0 (0.0) | 1 (1.2) | 0 (0.0) | 0 (0.0) | 0 (0.0) |
| None | 0 (0.0) | 1 (0.6) | 2 (2.4) | 0 (0.0) | 0 (0.0) | 1 (1.2) | 0 (0.0) | 1 (2.7) | 1 (2.2) |
| *How often do you eat meals that are purchased outside of the home, n (%)* | | | | | | | | | |
| Every day | 1 (1.1)* | 3 (1.7)*^¥^ | 1 (1.2)^¥^ | 1 (2.4)^‡^ | 0 (0.0)^‡^ | 2 (2.4)^§^ | 1 (1.0)^§^ | 1 (2.7)^#^ | 0 (0.0)^#^ |
| 4-6 times per week | 4 (4.4) | 9 (5.0) | 3 (3.7) | 3 (7.1) | 1 (2.1) | 5 (6.0) | 4 (4.2) | 3 (8.1) | 0 (0.0) |
| 2-3 times per week | 13 (14.4) | 11 (6.1) | 6 (7.3) | 6 (14.3) | 7 (14.6) | 7 (8.3) | 4 (4.2) | 6 (16.2) | 0 (0.0) |
| Once a week or less | 42 (46.7) | 67 (37.2) | 54 (65.9) | 25 (59.5) | 17 (35.4) | 40 (47.6) | 27 (28.1) | 23 (62.2) | 31 (68.9) |
| Never | 30 (33.3) | 90 (50.0) | 18 (22.0) | 7 (16.7) | 23 (47.9) | 30 (35.7) | 60 (62.5) | 4 (10.8) | 14 (31.1) |
| *Have you experienced a loss of appetite in the past month?* | 70 (77.8)*† | 13 (7.2)* | 2 (2.4)† | 33 (78.6) | 37 (77.1) | 4 (4.8) | 9 (9.4) | 0 (0.0) | 2 (4.4) |
| Do you observe fasting, n (%) | 40 (44.4)* | 107 (59.4)* | 43 (52.4) | 12 (28.6)^‡^ | 28 (58.3)^‡^ | 39 (46.4)^§^ | 68 (70.8)^§^ | 13 (35.1)^#^ | 30 (66.7)^#^ |
| *(Fasters only) How frequently do you fast, n (%)* | | | | | | | | | |
| Once or twice a week | 7 (17.5) | 21 (19.6) | 11 (25.6) | 2 (16.7) | 5 (17.9) | 5 (12.8) | 16 (23.5) | 3 (23.1) | 8 (26.7) |
| For a specified period of year | 33 (82.5) | 86 (80.4) | 32 (74.4) | 10 (83.3) | 23 (82.1) | 34 (87.2) | 52 (76.5) | 10 (76.9) | 22 (73.3) |
| *(Fasters only) What period of the year do you fast, n (%)* | | | | | | | | | |
| Ramadan | 24 (60.0) | 54 (50.5) | 15 (34.9) | 9 (75.0) | 15 (53.6) | 22 (56.4) | 32 (47.1) | 4 (30.8) | 11 (36.7) |
| Paryushan | 0 (0.0) | 0 (0.0) | 1 (2.3) | 0 (0.0) | 0 (0.0) | 0 (0.0) | 0 (0.0) | 0 (0.0) | 1 (3.3) |
| Other | 9 (22.5) | 32 (29.9) | 16 (37.2) | 1 (8.3) | 8 (28.6) | 12 (30.8) | 20 (29.4) | 6 (46.2) | 10 (33.3) |
| *(Fasters only) What best describes your fasting, n (%)* | | | | | | | | | |
| Consume only fruits, milk, sago, root vegetables, and flour throughout the day | 13 (32.5)^†^ | 51 (47.7)^¥^ | 27 (62.8)^†¥^ | 3 (25.0) | 10 (35.7) | 17 (43.6) | 34 (50.0) | 7 (53.8) | 20 (66.7) |
| Abstain from all foods and liquids during the day | 3 (7.5) | 1 (0.9) | 2 (4.7) | 0 (0.0) | 3 (10.7) | 0 (0.0) | 1 (1.5) | 0 (0.0) | 2 (6.7) |
| Abstain from food but not liquids during the day | 0 (0.0) | 2 (1.9) | 2 (4.7) | 0 (0.0) | 0 (0.0) | 1 (2.6) | 1 (1.5) | 2 (15.4) | 0 (0.0) |
| Follow the protocol of fasting for Ramadan | 24 (60.0) | 53 (49.5) | 12 (27.9) | 9 (75.0) | 15 (53.6) | 21 (53.8) | 32 (47.1) | 4 (30.8) | 8 (26.7) |
| Follow the protocol for Paryushan | 0 (0.0) | 0 (0.0) | 0 (0.0) | 0 (0.0) | 0 (0.0) | 0 (0.0) | 0 (0.0) | 0 (0.0) | 0 (0.0) |
| *Are you on any special diet, n (%)* | | | | | | | | | |
| Diabetic diet | 3 (3.3) | 2 (1.1) | 1 (1.2) | 2 (4.8) | 1 (2.1) | 2 (5.1) | 0 (0.0) | 1 (7.7) | 0 (0.0) |
| Low fat diet | 0 (0.0) | 0 (0.0) | 0 (0.0) | 0 (0.0) | 0 (0.0) | 0 (0.0) | 0 (0.0) | 0 (0.0) | 0 (0.0) |
| Low salt diet | 0 (0.0) | 0 (0.0) | 0 (0.0) | 0 (0.0) | 0 (0.0) | 0 (0.0) | 0 (0.0) | 0 (0.0) | 0 (0.0) |
| Weight-loss diet | 0 (0.0) | 1 (0.6) | 0 (0.0) | 0 (0.0) | 0 (0.0) | 0 (0.0) | 1 (1.5) | 0 (0.0) | 0 (0.0) |
| Others | 0 (0.0) | 1 (0.6) | 0 (0.0) | 0 (0.0) | 0 (0.0) | 1 (2.6) | 0 (0.0) | 0 (0.0) | 0 (0.0) |
| None | 87 (96.7) | 176 (97.8) | 81 (98.8) | 40 (95.2) | 47 (97.9) | 81 (207.7) | 95 (139.7) | 36 (276.9) | 45 (150.0) |
| Do you ever drink alcohol, including beer, n (%) | 19 (21.1) | 25 (13.9) | 15 (18.3) | 19 (45.2)^‡^ | 0 (0.0)^‡^ | 25 (29.8)^§^ | 0 (0.0)^§^ | 15 (40.5)^#^ | 0 (0.0)^#^ |
| *(Drinkers only) When you drink alcohol, what do you usually drink?* | | | | | | | | | |
| Country liquor | 3 (15.8) | 3 (12.0) | 3 (20.0) | 3 (15.8) | 0 (0.0) | 3 (12.0) | 0 (0.0) | 3 (20.0) | 0 (0.0) |
| English liquor | 8 (42.1) | 10 (40.0) | 6 (40.0) | 8 (42.1) | 0 (0.0) | 10 (40.0) | 0 (0.0) | 6 (40.0) | 0 (0.0) |
| Beer/wine | 8 (42.1) | 12 (48.0) | 6 (40.0) | 8 (42.1) | 0 (0.0) | 12 (48.0) | 0 (0.0) | 6 (40.0) | 0 (0.0) |
| Taddy/maddy | 0 (0.0) | 0 (0.0) | 0 (0.0) | 0 (0.0) | 0 (0.0) | 0 (0.0) | 0 (0.0) | 0 (0.0) | 0 (0.0) |
| Others | 0 (0.0) | 0 (0.0) | 0 (0.0) | 0 (0.0) | 0 (0.0) | 0 (0.0) | 0 (0.0) | 0 (0.0) | 0 (0.0) |
| *(Drinkers only) How often would you estimate that you drink alcohol, n (%)* | | | | | | | | | |
| Every day | 7 (36.8) | 4 (16.0) | 3 (20.0) | 7 (36.8) | 0 (0.0) | 4 (16.0) | 0 (0.0) | 3 (20.0) | 0 (0.0) |
| 4-5 days/week | 1 (5.3) | 1 (4.0) | 4 (26.7) | 1 (5.3) | 0 (0.0) | 1 (4.0) | 0 (0.0) | 4 (26.7) | 0 (0.0) |
| 1-3 days/week | 4 (21.1) | 2 (8.0) | 3 (20.0) | 4 (21.1) | 0 (0.0) | 2 (8.0) | 0 (0.0) | 3 (20.0) | 0 (0.0) |
| Less than once/week | 7 (36.8) | 18 (72.0) | 5 (33.3) | 7 (36.8) | 0 (0.0) | 18 (72.0) | 0 (0.0) | 5 (33.3) | 0 (0.0) |
| *Are you currently taking any nutritional supplements or tonics for over 1 week, n (%) (multiple responses allowed)* | | | | | | | | | |
| Vitamin D | 1 (1.1) | 0 (0.0) | 1 (1.2) | 0 (0.0) | 1 (2.1) | 0 (0.0) | 0 (0.0) | 1 (2.7) | 0 (0.0) |
| Calcium | 0 (0.0) | 0 (0.0) | 2 (2.4) | 0 (0.0) | 0 (0.0) | 0 (0.0) | 0 (0.0) | 1 (2.7) | 1 (2.2) |
| Multivitamin | 5 (5.6)* | 1 (0.6)* | 0 (0.0) | 3 (7.1) | 2 (4.2) | 0 (0.0) | 1 (1.0) | 0 (0.0) | 0 (0.0) |
| Other | 6 (6.7)* | 2 (1.1)* | 1 (1.2) | 3 (7.1) | 3 (6.3) | 1 (1.2) | 1 (1.0) | 1 (2.7) | 0 (0.0) |

**Footnote:** p for difference <0.05 (t-test for continuous variables, Fisher’s exact test for categorical variables): * cases vs. HH controls, † cases vs. community controls, ¥ HH vs. community controls, ‡ male vs. female cases, § male vs. female HH controls, # male vs. female community controls. Abbreviation: HH, household.
