# Supplementary material for: Diet and nutrition status of adult multidrug-resistant tuberculosis cases, household controls, and community controls in Mumbai, India
Source: PLOS Glob Public Health. 2026 Jan 13;6(1):e0005778. doi: 10.1371/journal.pgph.0005778 (PMC12798980; doi:10.1371/journal.pgph.0005778)
Supplement: S2 Table — (DOCX) [file pgph.0005778.s002.docx]

S2 Table. Estimated marginal mean (95%CI) consumption of key food groups and nutrients, and diet metric scores by age category and asset quartile.

| Dietary component |  | Age category |  |  |  | Asset Index quartile | |  | |
| --- | --- | --- | --- | --- | --- | --- | --- | --- | --- |
|  | <25 | 25-<40 | ≥40 | p-trend | 1 (lowest) | 2 | 3 | 4 (highest) | p-trend |
| *Food groups (g/day)* |  |  |  |  |  |  |  |  |  |
| Fruits | 154.0 (123.1-184.9) | 132.7 (102.7-162.7) | 110.2 (80.9-139.5) | 0.047 | 94.9 (60.8-128.9) | 134.9 (100.7-169.2) | 121.2 (87.0-155.3) | 174.8 (140.5-209.0) | 0.004 |
| Non-tuberous vegetables | 318.4 (285.5-351.3) | 307.4 (275.4-339.3) | 232.7 (201.4-263.9) | <0.001 | 232.5 (196.2-268.9) | 283.9 (247.4-320.4) | 301.1 (264.7-337.5) | 319.7 (283.2-356.2) | <0.001 |
| Nuts and seeds | 8.8 (7.5-10.1) | 7.7 (6.4-9.0) | 5.9 (4.7-7.1) | 0.002 | 4.8 (3.4-6.2) | 7.5 (6.1-8.9) | 7.7 (6.3-9.2) | 9.7 (8.2-11.1) | <0.001 |
| White roots and tubers | 23.2 (19.8-26.7) | 18.6 (15.3-21.9) | 12.1 (8.8-15.3) | <0.001 | 13.6 (9.8-17.4) | 15.6 (11.8-19.4) | 19.5 (15.8-23.3) | 22.2 (18.4-26.0) | <0.001 |
| Grains and flour products | 673.2 (603.2-743.3) | 660.4 (592.5-728.4) | 496.6 (430.2-563.1) | <0.001 | 578.9 (501.7-656.2) | 602.9 (525.3-680.4) | 609.6 (532.2-687.0) | 633.8 (556.3-711.4) | 0.330 |
| Animal-source foods | 343.3 (291.5-395.1) | 362.8 (312.5-413.1) | 334.6 (285.4-383.8) | 0.804 | 299.9 (242.8-357.1) | 336.0 (278.6-393.4) | 351.2 (293.9-408.5) | 399.4 (342.0-456.9) | 0.016 |
| Juice and SSBs | 36.9 (23.7-50.0) | 26.7 (13.9-39.4) | 23.6 (11.1-36.1) | 0.159 | 24.6 (10.1-39.2) | 19.0 (4.4-33.6) | 22.5 (8.0-37.1) | 49.0 (34.4-63.5) | 0.023 |
| Sweets and caloric sweeteners | 62.5 (54.4-70.7) | 44.9 (37.0-52.8) | 37.8 (30.1-45.5) | <0.001 | 41.1 (32.2-50.1) | 42.1 (33.1-51.1) | 49.5 (40.5-58.5) | 59.1 (50.1-68.1) | 0.003 |
| Oils | 52.1 (46.0-58.2) | 52.3 (46.4-58.2) | 42.3 (36.5-48.1) | 0.024 | 35.5 (28.7-42.2) | 47.2 (40.4-53.9) | 52.9 (46.1-59.6) | 59.2 (52.5-66.0) | <0.001 |
| Purchased deep fried foods | 58.1 (49.4-66.7) | 48.0 (39.6-56.4) | 31.1 (22.9-39.3) | <0.001 | 34.4 (24.9-44.0) | 37.8 (28.2-47.4) | 47.2 (37.7-56.8) | 61.2 (51.7-70.8) | <0.001 |
| *Nutrients* |  |  |  |  |  |  |  |  |  |
| Energy (kcal) | 2435 (2217-2653) | 2302 (2091-2514) | 1790 (1584-1997) | <0.001 | 1759 (1518-1999) | 2098 (1857-2340) | 2264 (2024-2505) | 2527 (2286-2769) | <0.001 |
| Protein (g) | 69.9 (62.0-77.7) | 70.1 (62.5-77.7) | 55.7 (48.3-63.1) | 0.011 | 50.5 (41.9-59.2) | 64.9 (56.3-73.6) | 68.0 (59.3-76.6) | 76.3 (67.6-84.9) | <0.001 |
| Saturated fat (g) | 21.3 (18.9-23.6) | 18.7 (16.4-21.0) | 14.9 (12.7-17.1) | <0.001 | 13.5 (10.9-16.1) | 17.0 (14.4-19.6) | 18.9 (16.3-21.5) | 23.2 (20.6-25.9) | <0.001 |
| Monounsaturated fat (g) | 18.9 (16.8-20.9) | 17.5 (15.5-19.4) | 14.0 (12.0-15.9) | <0.001 | 12.3 (10.0-14.5) | 15.9 (13.7-18.2) | 17.8 (15.5-20.0) | 20.7 (18.5-23.0) | <0.001 |
| Polyunsaturated fat (g) | 33.1 (29.0-37.2) | 33.2 (29.2-37.2) | 27.0 (23.1-30.9) | 0.037 | 21.8 (17.3-26.3) | 29.7 (25.2-34.3) | 34.1 (29.5-38.6) | 38.2 (33.7-42.7) | <0.001 |
| Alcohol (g) | -0.1 (-2.0-1.9) | 4.0 (2.1-6.0) | 4.2 (2.3-6.0) | 0.003 | 2.4 (0.3-4.6) | 2.5 (0.3-4.7) | 2.1 (-0.1-4.3) | 4.1 (2.0-6.3) | 0.348 |
| Fiber (g) | 9.7 (8.7-10.7) | 9.5 (8.6-10.4) | 7.4 (6.4-8.3) | <0.001 | 6.8 (5.7-7.9) | 8.4 (7.3-9.5) | 9.4 (8.3-10.5) | 10.6 (9.5-11.7) | <0.001 |
| Calcium (mg) | 756.7 (648.5-864.8) | 777.8 (672.8-882.8) | 670.7 (568.0-773.4) | 0.258 | 561.8 (442.5-681.2) | 706.5 (586.6-826.3) | 772.6 (653.0-892.2) | 891.6 (771.7-1011.4) | <0.001 |
| Iodine (μg) | 117.2 (103.8-130.6) | 113.1 (100.0-126.1) | 92.9 (80.2-105.7) | 0.011 | 90.5 (75.6-105.3) | 108.7 (93.9-123.6) | 108.0 (93.2-122.9) | 121.6 (106.8-136.5) | 0.006 |
| Iron (mg) | 16.5 (14.5-18.6) | 17.2 (15.2-19.2) | 14.0 (12.1-16.0) | 0.088 | 11.8 (9.5-14.1) | 15.4 (13.1-17.6) | 16.7 (14.4-19.0) | 19.5 (17.2-21.8) | <0.001 |
| Potassium (mg) | 2297.5 (2075.1-2519.8) | 2274.6 (2058.8-2490.5) | 1837.5 (1626.5-2048.6) | 0.004 | 1738.2 (1492.8-1983.6) | 2025.7 (1779.3-2272.0) | 2198.7 (1952.8-2444.5) | 2543.9 (2297.6-2790.2) | <0.001 |
| Selenium (μg) | 103.6 (88.4-118.8) | 113.5 (98.8-128.2) | 91.9 (77.5-106.3) | 0.269 | 82.3 (65.5-99.0) | 101.9 (85.1-118.7) | 105.9 (89.2-122.7) | 120.7 (103.9-137.5) | 0.002 |
| Zinc (mg) | 9.1 (8.3-10.0) | 9.1 (8.2-9.9) | 7.0 (6.2-7.8) | <0.001 | 6.8 (5.8-7.7) | 8.3 (7.3-9.2) | 8.8 (7.8-9.7) | 9.6 (8.7-10.6) | <0.001 |
| Vitamin A (μg RAE) | 952.6 (822.2-1083.0) | 843.6 (717.0-970.1) | 682.5 (558.8-806.3) | 0.004 | 595.2 (451.4-739.1) | 743.6 (599.1-888.0) | 860.1 (716.0-1004.2) | 1084.1 (939.7-1228.6) | <0.001 |
| Thiamine (mg) | 1.2 (1.1-1.3) | 1.2 (1.1-1.3) | 0.9 (0.8-1.0) | <0.001 | 0.9 (0.8-1.0) | 1.1 (0.9-1.2) | 1.1 (1.0-1.2) | 1.3 (1.2-1.4) | <0.001 |
| Folate (μg) | 321.5 (293.2-349.8) | 298.7 (271.2-326.2) | 222.1 (195.2-249.0) | <0.001 | 222.0 (190.8-253.3) | 272.4 (241.1-303.8) | 289.6 (258.3-320.9) | 330.7 (299.3-362.0) | <0.001 |
| Vitamin B12 (μg) | 2.0 (1.5-2.6) | 2.7 (2.1-3.2) | 2.5 (1.9-3.1) | 0.287 | 1.9 (1.2-2.5) | 2.1 (1.4-2.7) | 2.8 (2.1-3.4) | 2.9 (2.3-3.6) | 0.010 |
| Vitamin D (μg) | 1.0 (0.8-1.2) | 0.8 (0.7-1.0) | 0.7 (0.5-0.9) | 0.017 | 0.8 (0.6-1.0) | 0.8 (0.6-1.0) | 0.8 (0.6-1.0) | 1.0 (0.8-1.2) | 0.187 |
| *Diet metrics* |  |  |  |  |  |  |  |  |  |
| GDQS | 23.6 (22.8-24.3) | 24.0 (23.2-24.7) | 23.0 (22.2-23.7) | 0.253 | 21.8 (20.9-22.6) | 23.3 (22.4-24.1) | 23.7 (22.9-24.6) | 25.1 (24.3-26.0) | <0.001 |
| GDQS, energy-adjusted | 23.0 (21.7-24.2) | 24.1 (22.9-25.3) | 23.3 (22.2-24.5) | 0.681 | 21.1 (19.7-22.5) | 23.3 (21.9-24.6) | 23.7 (22.3-25.1) | 25.9 (24.5-27.3) | <0.001 |
| GDQS+ | 13.1 (12.2-13.9) | 13.1 (12.2-13.9) | 11.7 (10.8-12.5) | 0.020 | 10.5 (9.6-11.5) | 12.2 (11.2-13.1) | 12.8 (11.8-13.7) | 14.7 (13.8-15.7) | <0.001 |
| GDQS+, energy-adjusted | 12.7 (11.4-14.0) | 13.1 (11.9-14.3) | 11.9 (10.7-13.1) | 0.379 | 9.8 (8.4-11.2) | 12.0 (10.6-13.4) | 12.7 (11.3-14.1) | 15.7 (14.3-17.1) | <0.001 |
| GDQS- | 10.5 (10.2-10.8) | 10.9 (10.7-11.2) | 11.3 (11.0-11.6) | <0.001 | 11.3 (10.9-11.6) | 11.1 (10.8-11.4) | 10.9 (10.6-11.2) | 10.4 (10.1-10.7) | <0.001 |
| GDQS-, energy-adjusted | 10.3 (9.8-10.8) | 11.0 (10.5-11.5) | 11.4 (10.9-11.9) | 0.001 | 11.3 (10.8-11.9) | 11.2 (10.7-11.8) | 11.0 (10.4-11.6) | 10.2 (9.6-10.7) | 0.005 |

Footnote: Estimates are predicted values based on linear regression models adjusted for age category, asset quartile, sex, and case status. P for trend. Energy-adjusted metrics are computed using the residual method [[42]](#_bookmark41) (the mean metric score within each subgroup is added to the residuals for interpretability). Abbreviations: SSBs, sugar-sweetened beverages; RAE, retinol activity equivalents; GDQS, Global Diet Quality Score; GDQS+, GDQS positive sub-metric; GDQS-, GDQS negative sub-metric.

P <0.05 for interaction between household control status and age for: (i) consumption of nuts and seeds (ii) energy, and (iii) saturated fat.

P <0.05 for interaction between household control status and asset quartile for consumption of juice and sugar-sweetened beverages.

P< 0.05 for interaction between household control status and sex for (i) sweets and caloric sweeteners and (ii) alcohol.
