# Supplementary material for: Diet and nutrition status of adult multidrug-resistant tuberculosis cases, household controls, and community controls in Mumbai, India
Source: PLOS Glob Public Health. 2026 Jan 13;6(1):e0005778. doi: 10.1371/journal.pgph.0005778 (PMC12798980; doi:10.1371/journal.pgph.0005778)
Supplement: S1 Checklist — (DOCX) [file pgph.0005778.s003.docx]

Inclusivity in global research

PLOS’ policy on inclusivity in global research aims to improve transparency in the reporting of research performed outside of researchers’ own country or community and ensures that PLOS publications reporting global research adhere to high standards for research ethics and authorship. Authors of relevant research articles may be asked to complete the questionnaire below, which outlines ethical, cultural, and scientific considerations specific to inclusivity in global research. This questionnaire may be requested when researchers have travelled to a different country to conduct research, if research uses samples collected in another country, research with Indigenous populations or their lands, or if research is on cultural artefacts. Researchers travelling to another country solely to use laboratory equipment will not normally be required to complete the questionnaire. However, the questionnaire can be requested at the journal’s discretion for any submission – if you have been requested to complete this questionnaire by the PLOS journal you submitted to, please do so.

Please complete the questionnaire below and include this as a Supporting Information file with your manuscript. Note that if your paper is accepted for publication, this checklist will be published with your article in the supporting information files. Please ensure that you reference the checklist in the main body of your manuscript. We suggest adding a subsection ‘Inclusivity in global research’ to your Methods section and adding the following sentence: “Additional information regarding the ethical, cultural, and scientific considerations specific to inclusivity in global research is included in the Supporting Information (SX Checklist)”

The questions have been designed to be applicable to a wide range of study types, and there are subsections for both human subjects research and non-human subjects research. If any of the questions are not relevant to your research please mark them as “N/A” as appropriate.

**Ethical considerations, permits and authorship**

*This section is applicable to all research types.*

Provide details as to who granted permissions and/or consent for the study to take place in the Methods section of your manuscript. This should include the names of **all** ethics boards, governmental organizations, community leaders or other bodies that provided approval for the study. If individuals provided approval refer to these people by their role or title but do not list their name(s).

Reported on page number: 06, in the Methods section under the sub-section “Study population”. The study protocol was approved by the Harvard T.H. Chan School of Public Health Institutional Review Board (#IRB19-0237), Foundation for Medical Research Institutional Research Ethics Committee (#FMR/IREC/TB/01/2019), and Health Ministry’s Screening Committee of the Indian Council for Medical Research (# 2019-7974). I have uploaded original approval documents from all three committees in English.

If there were any deviations from the study protocol after approval was obtained please provide details of these changes in the Methods section of your manuscript.

Reported on page number: N/A

Did this study involve local collaborators that are residents of the country where the research was conducted or members of the community studied? If you do not have any authors from said communities, please provide an explanation for this below.

Yes. This study was conducted under a collaboration between the Harvard T.H. Chan School of Public Health and the Foundation for Medical Research – which is a recognized national research organisation by the Government of India. Multiple co-authors of this study are based at FMR (or were affiliated with FMR at the time of the study implementation)- including the first (LG) and last authors (NM). Furthermore, the study involved local collaborators from **LSS (Lok Seva Sangam)**, a community-based NGO based in M-ward of Mumbai, India where the research was conducted. LSS provided trained field workers who supported data collection and logistical coordination. While members of LSS were not listed as co-authors as they did not meet all criteria for authorship, their contributions were essential to the successful implementation of the study and are acknowledged appropriately.

Everyone listed as an author should meet PLOS’ criteria for authorship and all individuals who meet these criteria should be included in the author byline, rather than the acknowledgements. For further information please see the journal’s Authorship Policy.

**Human subjects research (e.g. health research, medical research, cross-cultural psychology)**

Did you obtain written informed consent from a representative of the local community or region before the research took place? How did you establish who speaks for the community? Details of written informed consent obtained from study participants should be reported separately in the Methods section of your manuscript.

We obtained all required ethical and regulatory approvals required in India before undertaking the study. Written informed consent from individual study participants was obtained before their enrolment in the study as described in the Methods section. Community-level permission was also sought through consultation with the local department of tuberculosis health authorities, who were identified based on their formal roles in local public health outreach. No separate written community consent was required beyond these established procedures. The details on permissions acquired for the study are mentioned in Methods section of the manuscript.

How did members of the local community provide input on the aims of the research investigation, its methodology, and its anticipated outcome(s)?

Input from the local community was gathered through discussions with community health workers and local health officials during the study planning phase. Their insights helped refine the research aims, ensure that the methodology was contextually appropriate, and align the anticipated outcomes with local health priorities and concerns.

Informed consent documents and related study materials were translated into the local language by staff fluent in the local language, who also reviewed for cultural relevance and clarity. Trained study staff fluent in the local language conducted the consent process verbally to ensure participant understanding, and participants were encouraged to ask questions before providing informed written consent.

When engaging with the local community, how did you ensure that the informed consent documents and other materials could be understood by local stakeholders?

Will the findings of the research be made available in an understandable format to stakeholders in the community where the study was conducted (e.g. via a presentation, summary report, copies of publications, etc.)? Please provide details of how this will be achieved.

Yes, the findings of the research will be shared with stakeholders in the community where the study was conducted. This will be achieved through summary reports in the local language, presentations of findings to regional health authorities, and by providing copies of any resulting publications to relevant institutions involved in the study.

**Non-human subjects research using specimens/ animals collected as part of the study, or those housed in archival collections. Examples include archaeology, paleontology, botany and zoology.**

Did the permission you obtained from a local authority to perform the study include an agreement on access to outputs and benefit sharing? This may include procedures to enable fair distribution of the benefits and resources arising from the research performed. Please include any details of Prior Informed Consent and Benefit Sharing Agreements obtained. These may be required by field-specific regulations, for example the Convention on Biological Diversity (CBD) and the associated Nagoya Protocol.

N/A: This is not applicable, as the study involved human subjects in the research and did not involve the collection or use of non-human specimens or materials.

If the material used in your study was imported, please A) provide the year it was imported and B) indicate whether permits were obtained to import/export the materials used, C) provide details of any permits obtained. If this information is not available, please indicate this.

N/A

If you used archival specimens, please state how the material used in your study was acquired by the institute it is held in and provide details of any permits obtained for the original excavations/ sample collection. If this information is not available, please indicate this.

N/A: This study did not use any archival specimens, and no materials from previous excavations or collections were analyzed.

How was the potential cultural significance of the materials collected in your study to local communities considered in your research design? Were Indigenous peoples and/or local researchers and institutions involved with archaeological excavations / collection of specimens? If so, please provide a description of their involvement.

N/A: No culturally significant materials were collected, and no archaeological excavations or specimen collections were conducted as part of this study.

If your manuscript includes photographs of human remains please indicate whether authors obtained permission from descendants or affiliated cultural communities to do so.

N/A: No photographs of human remains were included in this research.
