# Supplementary material for: Diet and nutrition status of adult multidrug-resistant tuberculosis cases, household controls, and community controls in Mumbai, India
Source: PLOS Glob Public Health. 2026 Jan 13;6(1):e0005778. doi: 10.1371/journal.pgph.0005778 (PMC12798980; doi:10.1371/journal.pgph.0005778)
Supplement: Attachment — Submitted filename: Response to Reviews.docx [file pgph.0005778.s005.docx]

**Diet and nutrition status of adult multidrug-resistant tuberculosis cases, household controls, and community controls in Mumbai, India (PGPH-D-24-02874)**

**Response to Reviewers’ Comments**

(Page and Line numbers refer to “Revised Manuscript with Track Changes”)

**Reviewer #1:**

**Introduction:**

**1. The introduction is overall well-written; however, for further improvement, it is recommended to:**

a) **Combine or merge the first and second paragraphs for better flow and coherence.**

Response: Thank you for this suggestion. We have now combined both paragraphs as suggested. Page references for updated text are noted below (page numbers refer to the updated, tracked manuscript).

Introduction, page:04

b) **Begin by addressing the global burden of the problem and place the global context in the first paragraph to set the stage effectively.**

Response: We thank the Reviewer for this comment, and agree that some global contextualization may be helpful. We have therefore revised the beginning of the Introduction with relevant figures to highlight the global context.

Introduction, page:04

c) **Additionally, please ensure that findings from previous studies are reported accurately as presented in the original papers. For example, in lines 47 and 48 of the introduction, the author reported the risk of TB in urban residents as 1.56 (referencing paper no. 3). However, the original paper indicates that the risk is 1.58. Please revise this and carefully cross-check references throughout the manuscript.**

Response: We thank the Reviewer for catching this. Referring back to paper no.3, we can indeed see that the adjusted risk ratio is 1.589 (1.036–2.437). We have therefore amended the relevant statement in the Introduction. We have also double-checked the other figures cited throughout.

Introduction, page:04, line 48-49

**2. Methodology:**

**The methodology section is somewhat unclear and lacks precision. It is recommended to:**

a) **Present the methods in a clearer and more structured manner.**
b) **Avoid jumping quickly from one point to another, as this may confuse readers and reduce their motivation to continue reading.**

Response: We understand the Reviewer’s point that the current version of the Methods could be further simplified for ease of reading, including smoother transitions from one point to another. We have edited the section in line with this advice, including clear sequencing of sub-sections.

Methods, pages: 05-12

**3. Results:**

**The results section would benefit from starting with a summary of the characteristics of the study participants. This should include key geographic, demographic, and socioeconomic details to provide the audience with a better understanding of the study population.**

Response: We appreciate the Reviewer’s point, and have now added information regarding the study population details into an initial paragraph in the Results.

Results, page: 13, lines: 286-293

**4. Tables:**

**To improve readability, it is recommended to link the discussion in the paragraphs with the corresponding tables. This will allow readers to easily navigate back and forth between the text and the tables.**

Response: Thank you for this comment. We have now made sure to include the Table reference at the end of each sentence in the Results and Discussion sections to enable easier referencing by the reader.

**Reviewer #2:**

**Summary**

**5. The authors used data from a case-control study to explore the nutritional trends among MDR-TB cases in comparison to household and community controls. The authors report significant differences in nutritional status and dietary consumption between the cases and controls. I commend the authors for their undertaking of difficult nutritional assessments.**

Response: We thank the author for their review of this paper, and for this positive feedback.

**Major Issues:**

**6. Clarification and justification of methodological and statistical approaches is needed. The results section would benefit from streamlining and reorganization as presently comprehension is challenging.**

Response: We thank the Reviewer for this comment, and appreciate that further clarification of the methodological approaches and organization of the Results may be beneficial. We understand the Reviewer has noted specific comments below, and have listed our responses to each.

**Methods**

**7. Details are missing regarding timing and frequency of data collection.**

Response: Thank you for this comment. As this was a case-control study, data were collected only once for each participant, following enrolment. Data collection was conducted from January 28, 2020 to December 17, 2020. We have made this clearer in the Methods section.

Methods, page: 06-07

**Dietary assessment**

**8. Dietary assessment(s) is a fundamental part of this study, and as such each assessment should be sufficiently addressed. The authors mention the administration of a food frequency questionnaire; however, there is also mention of additional dietary assessments which are undefined. As currently written, it is unclear what nutritional data were collected, overall. There are minimal details on quantifying food consumption without stating how this data were initially collected.**

Response: We understand that the exact dietary assessments may not have been clear and comprehensive, and have now made an effort to address this in the Methods. Briefly, we conducted two key data collections related to food: (1) a semi-quantitative food-frequency questionnaire to understand and quantify what participants usually consumed over the past year, plus (2) a set of questions on food consumption-related habits, which sought to understand related practices at the household level, e.g. whether a participant fasted, used supplements or alcohol, and what sorts of oils or thickening agents were used to prepare food at the household level.

Methods, page: 07-08

**9. Lines 134-136. The authors allude to food consumption data, but it is unclear where these data come from. Further, authors fail to provide justification for a 2000 kcal/day threshold. Explanation of “dish-bashed” food groups is necessary as readers may be unfamiliar with what this means substantively and nutritionally. It is also unclear whether these food items were converted into g/day and/or g/2000 kcal/day.**

Response: Thank you for these notes. We have now rearranged the Methods section and added further details to make clearer the origin of the food consumption data. Specifically, we calculated food and food group intake, nutrient intake and adequacy, and diet metric scores using the food frequency questionnaire data.

Methods, page: 07-08

The justification for the 2000kcal/day threshold is that this is the mean intake of the population, as per an ICMR-NIN-based assessment (1). We have now made this clearer in the text.

Methods, page:09, line:185-187

Thanks also for identifying the error with the term “dish-bashed”. This was a typographical error, and the correct term is “dish-based”, i.e. food groups that are distinguished by local culinary practices instead of inherent nutritional significance. We have now made this clearer in the text.

Methods, page 09, line: 185-186

To confirm, we quantified to both g/day as well as g/2000kcal/day, as outlined in the Methods, and also in the Results, Table 2.

Methods, page 09: text: **“Food consumption data were used to calculate consumption of key food groups in g/day and g/2000 kcal/day (to aid comparisons across subgroups, with the cutoff of 2000 kcal/day based on national estimates of mean daily caloric intake, and consumption of a separate set of “dish-based” food groups (food groups that are distinguished by local culinary practices instead of inherent nutritional significance)…..”**, and Results, page: 13-14 under the sub-section, **“Consumption and consumption density of key food groups”**

**10. Lines 144-146. The inclusion of phytochemical index (PI) is questionable. The manuscript would benefit from elucidating the purpose PI serves nutritionally and in context to people living with MDR-TB. Presently, it is uncertain how this nutritional marker servers the aim of the paper and population of interest.**

Response: We agree with the Reviewer that the PI is less relevant to this analysis. We have therefore now removed it from the manuscript.

Methods, page 09, line: 194-197, and Results, page 14, table 3

**11. Lines 150-151. Authors state that food composition data was “supplemented as needed using data from the USDA and UK.” What was the justification for using two additional data sources and what data points were used from the USDA and UK, respectively?**

Response: We appreciate that the specific data points and rationale for supplementing food composition data was not entirely clear in the Methods. We have now corrected this. Briefly, we supplemented food composition data only for vitamin D, using information from the USDA, due to implausibly high results obtained regarding vitamin D concentrations for plant-source foods from the 2017 Indian Food Composition Table. There were no additional data points that were supplemented in this way, and none used information from the UK.

Methods, page 10

**12. Other assessments**

**Line 170. The manuscript states that household-food consumption related habits were collected, yet there is no indication of this assessment under dietary assessments and how these data were quantified. It is unclear whether the household measure is a distinct measure from the FFQ and other dietary assessments mentioned previously.**

Response: We thank the Reviewer for this comment. We agree that the meaning of household food consumption-related habits was not entirely clear. This point was also raised by another Reviewer, and in response, we have rearranged and added more detail regarding the household food consumption-related habits in the Methods.

Methods, page 07, under the sub-section: **Food consumption-related habits**

**Statistical analysis**

**13. Line 194-195. Authors state that an asset score was computed and a principal component analysis employed. However, justification for why a PCA analysis was conducted is missing. Further, there is little information regarding the asset index. The methods section would benefit from describing what type of asset index was calculated and how this variable was operationalized.**

Response: We agree with the Reviewer that some additional detail regarding the rationale, construction, and operationalization of the asset index variable would be helpful. In this study, we collected multiple measures of socioeconomic status (SES), including education, as well as assets available in the household (e.g. chair, table, pressure cooker, bicycle, etc.) (2). There is much discussion in the literature about the relative advantages and disadvantages of these aspects of SES (3). Asset index is considered particularly useful as an indication of longer-term SES of the household, and includes a combination of relevant information regarding availability of a range of assets in the household (3). Hence, we decided to examine the asset index as the SES indicator of choice when examining the distribution of diet- and nutrition-related variables. We constructed this variable using principal components analysis, which is a standard method that enables a statistical summarization of information from a large number of variables (4,5). We have now explained this in greater detail in the manuscript.

Methods, page: 11, line: 243-252

**14. Line 209-211. The manuscript details that regressions with interaction term(s) were conducted. It is unclear whether the authors are exploring a combined effect between variables or effect modification.**

Response: Thank you for this note. We can confirm that regressions with interaction terms were conducted in order to examine potential effect modification, and we have now made this clearer in the text.

Methods, page 12, line 273-274

**15. Additionally, models include a diet metric that was energy-adjusted via residual method. The authors do not comment on the purpose for or what this adjustment achieves.**

Response: We have now made clearer the purpose of energy adjustment in the manuscript. Specifically, energy adjustment is used to account for potential correlations between energy intake and specific nutrient intake, and understand variations in nutrient intake across the study population that are independent of total energy intake (6).

Methods, page 12, line 275-277

**Results**

**16. Overall, the results were difficult to follow, and key points were not easily identified. This section was extensive and would benefit from being pared down. Data was expressed in large tables though figures would showcase the data nicely and improve interpretability.**

Response: We thank the Reviewer for this note. We have now introduced changes to the Results section in order to make the key points easier to follow (including edits to make it shorter). We have also replaced Table 7 with a figure, and moved the table to Supplementary.

Results, page 13-18

**17. Lines 323-333 Reporting beta coefficients is less interpretable than reporting relative measures.**

Response: We note that the Reviewer’s comment is about descriptions of results related to Table 7 (“In adjusted interaction models, the main effects of household control status on consumption of nuts and seeds (β=5.5 g/day, p<0.001), […]”). While we understand that there may be a preference to see relative measures, there are important advantages to understanding first the absolute differences in food and nutrient intakes as represented by the beta coefficients. Given that beta coefficients represent absolute differences in nutrient or food intake in measurable units (e.g. g/day), these should be helpful to interpret, and important to understand. Furthermore, and importantly, examining continuous variables in this way enables preservation of statistical power (7). However, we understand the reviewer’s point, and in line with other comments to shorten the Results and make the key points more obvious, we have instead made a broader reference to our findings relating to effect modification (where the beta coefficients were first reported).

Results, page 13-18

**Discussion**

**18. The authors did an insufficient job at exploring how nutritional support will reduce burden of MDR-TB, specifically. It seems, rather, that nutritional support will benefit those with TB infection regardless of drug susceptibility. Perhaps authors should explore how poor nutrition exacerbates disease outcomes and how potential interventions can mitigate adverse outcomes among MDR-TB patients.**

Response: We appreciate the reviewer’s insightful comment and agree that further elaboration was warranted on the specific relevance of nutritional support in the context of multidrug-resistant tuberculosis (MDR-TB). In response, we have expanded our discussion to clarify the suggested point, drawing directly from the findings and implications of the RATIONS trial which was previously included in the discussion section.

Discussion, page: 19 & 22

**19. The justification for community controls is underexplored and not well articulated. Discussion is lacking on how community data support the research conclusions and help advance the field.**

Response: Thank you for this comment. In case-control studies, the approach to choosing a control group is to ensure that controls are drawn from the same population as cases with respect to the exposure of interest (8). A variety of control groups may be used, including family controls, community controls, and others. Each type of controls may have their own advantages and disadvantages (9). As noted in the manuscript, we used both household controls and community controls. We recruited community controls from non-respiratory departments at the hospitals where the cases were identified, and were matched with cases by age, sex, and ward of residence. In our case, household controls may have been appropriate to account for potential confounding due to socioeconomic status, but may present the risk of overmatching particularly with regards to shared environment and access to similar foods. On the other hand, the use of community controls overcomes this issue, and enables a more representative view of the broader population and increases generalizability of study findings (9). In any case, use of community controls affords a comprehensive examination of population level dietary habits in a ward with high TB prevalence in Mumbai. We have now discussed these relative advantages and disadvantages more in the Discussion.

Discussion, page 21-22

**20. This study was conducted during COVID-19 pandemic, and it is likely that the pandemic had direct impacts on food supply.**

Response: We agree with the Reviewer’s comment that the COVID-19 pandemic, which occurred during the course of data collection for this study, may have affected participants’ diet at the time of the data collection. However, importantly, the questionnaire specifically asked about participants’ habitual intake over the past year, and is therefore more representative of more long-term trends in diet. We agree that more concurrent problems in food supply may still have been a concern, but we expect that this would be small in terms of effects on reporting of usual diet in the past year. Nonetheless, we have included relevant statements regarding this in the Discussion.

Discussion, page 21

**21. Data availability**

**Data is accessible upon request. This does not meet the PLOS data policy.**

Response: Thank you for this comment. We fully agree with the principle underlying the PLOS data policy to make data as available as possible in the interests of transparency. However, our ability to make data available to other researchers must be in line with: (1) consent provided by participants regarding making their data publicly accessible, and (2) legal and regulatory permissions at the country level regarding making data publicly accessible. Due to these ethical and regulatory restrictions, we cannot make data publicly available at this time. However, we would be happy to discuss with any interested researchers who would like to access the data and explore setting up relevant legal and other arrangements to share data. This is in line with the relevant statements that are also noted in the journal’s guidelines regarding the data availability statement (<https://journals.plos.org/globalpublichealth/s/data-availability> ).

**Reviewer #3:**

**22. This study is a commendable effort on describing in detail the nutritional intake of MDR-TB patients in comparison with their household contacts (HHC) and the community controls in the urban slum of Mumbai, where the burden of TB is high. However, the major issue is the screening of HHC only for symptoms of TB and not for the presence of TB infection (TBI) or sub-clinical TB. Presence of clinical or sub-clinical TV may play a role in their nutritional status.**

Response: We thank the Reviewer for their comments, and for their time reviewing this manuscript. To clarify, we did screen household controls for the presence of TB infection via QFT-G IGRA (2). Using this method, we found that 36% of household controls and 32% of non-household controls were IGRA-positive (2). We agree that this may affected the nutritional intakes and status of the controls, which may have affected the magnitude of the differences observed between cases and controls. We have now made this clearer as a limitation in the Discussion section.

Methods: page 08.

Results: page 13.

Discussion, page 21-22.

**23. Other issue is regarding use analytical methods with multiple sub-groups with probably inadequate sample size for applying the statistical tests.**

Response: This study was a secondary analysis of a case-control study examining the role of vitamin D status as a potential risk factor for MDR-TB. Hence, power calculations were made on this basis, and are outlined in the protocol for this study (10). Nonetheless, we observed statistically significant differences between a range of diet- and nutrition-related indices and MDR-TB case or control status, as evidenced in the tables. We agree that in cases where associations were not observed, this may have been due to issues relating to sample size, and have now made this clearer in the Discussion.

Discussion, page 21.

**24. Overall the paper has many errors in spelling and language, which need to be corrected. Specific comments are as follows.**

**Introduction:**

**In the paragraph 1 lines 38-39, the number of cases of MDR-TB is mistakenly stated out of the proportion of infected.**

Response: Thank you for this comment. We are referring to figures that are reported by the World Health Organization (<https://worldhealthorg.shinyapps.io/tb_profiles/?_inputs_&tab=%22charts%22&lan=%22EN%22&iso2=%22IN%22&entity_type=%22country%22>). Within this, we are citing the figures “Estimated percentage of people with TB who had multidrug-resistant or rifampicin-resistant TB (MDR/RR-TB)” – which indeed indicates proportion of drug-resistant cases out of the proportion of infected. We have edited the text to make clearer the numerator and denominator (also noting that this included both MDR- and RR-TB).

Introduction: page 04.

**25. The information on the proportion of untreated cases is not essential for this paper.**

Response: Thank you for this comment. We have now removed the relevant figures from the Introduction.

Introduction: page 04, lines 46-47.

**26. Methods:**

**Was the patient group homogenous? It will be good to mention the resistance pattern and the mode of diagnosis.**

Response: Thank you for this comment. The patient group was relatively homogeneous by

sociodemographic characteristics – now outlined in more detail in the Results section.

Regarding diagnosis, we enrolled patients with confirmed MDR-TB directly from facilities with

the aid of facility staff, and abstracted relevant data regarding diagnosis and resistance patterns

from the hospital records. Unfortunately, however, abstracted data were relatively sparse

regarding diagnosis mode and resistance patterns, and hence we are not able to summarize

this in more detail. We have therefore added a statement to this effect in the limitations of the

manuscript – noting also that this does not materially impact the substance and focus of the

current analysis.

Results: page 13.

Discussion: page 21.

**27. Standard terms should be used instead of terms such as non-tuberculotic household controls. HHC of TB patients might be having TB infection and therefore cannot be called non-tuberculotic.**

Response: We agree with the Reviewer’s point, and have now removed the expression “non-tuberculotic”.

Methods: page 06, line: 92.

**28. Authors need to mention the validity of the method of estimating the quantity of food consumed by the use of Food Frequency Questionnaires. What is the percentage of error in the estimation of food consumption using this method?**

Response: Thank you for this comment. We have now added the validity of FFQ method in the manuscript. The validity of estimating food consumption using Food Frequency Questionnaires (FFQs) has been a subject of considerable evaluation. In the study by Bowen et al. (2011), the reproducibility and validity of the FFQ developed for the Indian Migration Study (IMS) which was adapted to be used in the present study were found to be comparable to those reported in other regional Indian FFQ validation studies (11). Consistent with findings from both Indian and Western contexts, the authors reported a tendency for FFQs to overestimate intake when compared to reference methods such as 24-hour dietary recalls or weighed food records (11).

Despite this tendency, the IMS FFQ demonstrated acceptable performance across diverse regions of India, suggesting robust applicability beyond localized populations. As Willett (1998) notes, correlation coefficients between FFQs and reference methods for nutrient intake typically range from 0.5 to 0.7, implying an error margin of approximately 20% to 40% in estimating absolute intake (12). However, FFQs remain valuable tools in epidemiological studies due to their ability to effectively rank individuals by intake, even if precise quantities are less accurate.

In summary, while FFQs may introduce some degree of measurement error, the validated IMS FFQ offers a reliable approach for assessing dietary intake patterns in diverse Indian populations.

Methods: page 07

**29. Why the BMI classification for Asian Indians was not used?**

Response: Thank you for catching this. We did indeed use the BMI classification for Asian Indians, and mis-noted this in the text. We have now made the relevant change in the text in the Methods, to correctly reflect that we used Asian Indian classifications.

Methods: page 11.

**30. Sample size estimation was not done. Was the sample size adequate for the multiple sub-group analysis (tertiles of age and quartiles of assets index as well as the nine groups stratified by sex)?**

Response: Thank you for this note. As mentioned earlier, this study was a secondary analysis of a case-control study examining the role of vitamin D status as a potential risk factor for MDR-TB. Hence, power calculations were made on this basis, and are outlined in the protocol for this study (10). Nonetheless, we observed statistically significant differences between a range of diet- and nutrition-related indices and MDR-TB case or control status, as evidenced in the tables. We agree that in cases where associations were not observed, this may have been due to issues relating to sample size, and have now made this clearer in the Discussion.

Discussion, page 21-22

**31. What was the rationale for categorising the age into <25, 25-40 and >40 years? How can the three parts of the tertiles be unequal?**

Response: We agree with the Reviewer that “tertiles” is not an accurate description of the age categories. Regarding the cutoffs for categories, 25 years is a common cutoff to distinguish young adulthood (13,14), while 40 years is often used to indicate the next phase of older adulthood (15). We have now removed references to “tertile” throughout the text.

**32. Why the variables used in the multi-variable model were only limited to age, asset index, sex, and study arm? There are multiple confounders such as alcohol/ tobacco use and other co-morbidities, especially HIV, which have not been considered in the analysis.**

Response: We thank the Reviewer for this important note. We adjusted for these key social and demographic variables only for two reasons. First, we were keen to understand the distribution of nutritional intakes specifically across these variables. Secondly, socioeconomic status (SES), as broadly represented by asset index, would likely influence health-related behaviors and status, including alcohol or tobacco use and disease status. Hence, these factors are more likely to be on the pathway from SES to nutritional intake, and therefore including them in regression models would likely represent over-adjustment (16). We have therefore not included them in models.

**33. Results: some of the results can be presented in graphs to reduce the lengthy texts.**

Response: Thank you for this note. We have now edited the Results section to make it easier to follow, and replaced results in Table 7 into graphs.

Results, page 17

**34. Discussion: limitations need to be added.**

Response: Thank you for this note. We have now more directly addressed the limitations in the Discussion section.

Discussion, page: 21-22

**References:**

1.	ICMR National Institute for Nutrition. What India Eats [Internet]. Hyderabad: ICMR-NIN; 2020 [cited 2025 Jul 8]. Available from: https://www.nin.res.in/nutrition2020/what_india_eats.pdf

2.	Shukla A, Bromage S, Dholakia Y, Hemler EC, Dev P, Govekar L, et al. Case-control study of vitamin D status and adult multidrug-resistant pulmonary TB. The International Journal of Tuberculosis and Lung Disease. 2022 Sep 1;26(9):826–34.

3.	Vyas S, Kumaranayake L. Constructing socio-economic status indices: how to use principal components analysis. Health policy and planning. 2006 Nov;21(6):459–68.

4.	The DHS Program - Wealth-Index-Construction [Internet]. [cited 2025 Jul 6]. Available from: https://dhsprogram.com/topics/wealth-index/Wealth-Index-Construction.cfm

5.	Murphy GA, Asiki G, Ekoru K, Nsubuga RN, Nakiyingi-Miiro J, Young EH, et al. Sociodemographic distribution of non-communicable disease risk factors in rural Uganda: a cross-sectional study. Int J Epidemiol. 2013 Dec;42(6):1740–53.

6.	Willett W, Stampfer MJ. Total energy intake: implications for epidemiologic analyses. Am J Epidemiol. 1986 Jul;124(1):17–27.

7.	Altman DG, Royston P. The cost of dichotomising continuous variables. BMJ. 2006 May 6;332(7549):1080.

8.	Moritz DJ, Kelsey JL, Grisso JA. Hospital Controls versus Community Controls: Differences in Inferences Regarding Risk Factors for Hip Fracture. American Journal of Epidemiology. 1997 Apr 1;145(7):653–60.

9.	Wacholder S, Silverman DT, McLaughlin JK, Mandel JS. Selection of controls in case-control studies. II. Types of controls. Am J Epidemiol. 1992 May 1;135(9):1029–41.

10.	Mistry N, Hemler EC, Dholakia Y, Bromage S, Shukla A, Dev P, et al. Protocol for a case–control study of vitamin D status, adult multidrug-resistant tuberculosis disease and tuberculosis infection in Mumbai, India. BMJ Open. 2020 Nov 1;10(11):e039935.

11.	Bowen L, Bharathi AV, Kinra S, Destavola B, Ness A, Ebrahim S. Development and evaluation of a semi-quantitative food frequency questionnaire for use in urban and rural India. Asia Pac J Clin Nutr. 2012;21(3):355–60.

12.	Willett WC. Nutritional Epidemiology [Internet]. Oxford University Press; 1998 [cited 2025 Jul 10]. Available from: https://doi.org/10.1093/acprof:oso/9780195122978.001.0001

13.	Patton GC, Sawyer SM, Santelli JS, Ross DA, Afifi R, Allen NB, et al. Our future: a Lancet commission on adolescent health and wellbeing. Lancet. 2016;387(10036):2423–78.

14.	Sawyer SM, Azzopardi PS, Wickremarathne D, Patton GC. The age of adolescence. The Lancet Child & Adolescent Health.

15.	Goodman-Palmer D, Greig C, Agyapong-Badu S, Witham MD, Payne CF, Bountogo M, et al. Frailty progression in adults aged 40 years and older in rural Burkina Faso: a longitudinal, population-based study. The Lancet Healthy Longevity. 2024 Jul 1;5(7):e493–502.

16.	Victora CG, Huttly SR, Fuchs SC, Olinto MT. The role of conceptual frameworks in epidemiological analysis: a hierarchical approach. Int J Epidemiol. 1997 Feb;26(1):224–7.
